# Periodontal precision: diagnostic skills and confidence of dentists in Asian countries in applying the 2017 EFP/AAP periodontal disease classification- a cross-sectional pilot study

**DOI:** 10.1186/s12903-025-07030-x

**Published:** 2025-12-05

**Authors:** Kamran Ali, Priti Charde, Daniel Zahra, Ewen McColl, Tayeb Al Hadeethi, Nader Hamdan

**Affiliations:** 1 College of Dental Medicine, QU Health, Qatar University, Doha, 2713 Qatar; 2 School of Psychology, Plymouth University, Plymouth, PL4 8AA UK; 3 Peninsula Dental School, Faculty of Health, University of Plymouth, Plymouth, UK; 4 Faculty of Medicine & Dentistry, University of Alberta, Edmonton, Canada

**Keywords:** Dentistry, dental education, Dentists, Guidelines, Periodontitis

## Abstract

**Background:**

The classification of periodontal disease published in 2017 by the European Federation of Periodontology (EFP) and American Academy of Periodontology (AAP), provides a framework for diagnosis and treatment. The aim of this study was to evaluate the diagnostic skills and self-perceived confidence of dentists and dental students based in Asian countries in the use of this classification.

**Methods:**

A cross-sectional analytic study design was employed. An online questionnaire encompassing four periodontitis cases was used for data collection. A total of 500 participants were invited to provide a diagnosis and rate their confidence for each case.

**Results:**

Responses were provided by 312 participants completed including 192 females and 120 males. Analysis of variance (ANOVA) showed a statistically significant difference in accuracy across cases by Professional Role (F (9,924) = 2.304, *p* = 0.005), and an overall difference on accuracy by Professional Role (F (1,308) = 2.304, *p* = 0.012). The diagnostic accuracy mean was highest for periodontics specialists (57.81 ± 49.78) followed by general dentists (50.00 ± 50.31), other dental specialists (45.00 ± 50.06); and dental students (25.00 ± 43.55). A statistically significant difference in confidence was noted across Age Groups, Gender, and Roles (*F*_(1,291)_ = 6.356, *p* < 0.001; *F*_(1,293)_ = 13.747, *p* < 0.001; *F*_(1,291)_ = 8.731, *p* < 0.001 respectively). There was no statistically significant effect on confidence ratings by any interaction between Location and Case.

**Conclusion:**

The study shows the diagnostic accuracy and confidence was highest amongst periodontology specialists followed by general dentists and undergraduate students. Overall the participants showed suboptimal diagnostic accuracy and confidence.

**Supplementary Information:**

The online version contains supplementary material available at 10.1186/s12903-025-07030-x.

## Introduction

Periodontal disease is most common oral health condition affecting about 45%–50% of world’s population [1]. It’s classification provides a framework for diagnosis and treatment planning of patients in a consistent and reliable manner [2]. With advancing knowledge of its pathogenesis and evidence-based therapies, its classification has been updated periodically during the past five decades by the Periodontology World Workshop by consensus amongst international experts [3].

Over the past five decades, the classification of periodontal diseases has undergone several revisions, reflecting advances in scientific understanding of their pathogenesis [4, 5]. Earlier frameworks, such as the 1999 Classification of Periodontal Diseases and Conditions, distinguished between “chronic” and “aggressive” periodontitis, categories largely defined by clinical presentation and disease progression [6]. 

Subsequent research on the pathogenesis of periodontal disease demonstrated substantial overlap between chronic and aggressive forms, while also underscoring the central role of the host immune-inflammatory response and modifying factors such as smoking and diabetes in disease susceptibility and outcomes [6]. This culminated in the 2017 World Workshop on the Classification of Periodontal and Peri-Implant Diseases and Conditions, which emphasized the multifactorial nature of periodontal disease, integrating clinical parameters with an improved appreciation of host susceptibility and response [6, 7]. This shift marked a significant step toward a more biologically driven framework, aligning diagnosis and treatment planning with contemporary evidence [3, 8]. The 2017 classification of periodontal disease by American Academy of Periodontology (AAP) and the European Federation of Periodontology (EFP), groups periodontitis into a multidimensional staging and grading system [7–9]. 

Since the 2017 classification system is considerably different from previous versions and incorporates several variables to diagnose periodontal disease, application in clinical practice has been challenging particularly for general dental practitioners (GDPs and dental students [9]. Many clinicians expressed their concern regarding its application in daily practice due to its complexity. Given that the initial diagnosis and treatment of periodontal disease is done by general dental practice settings, the role of general dental practitioner is crucial and warrants a good understanding and confidence to apply this classification. This is important to inform clinical decision-making by general practitioners regarding prevention, diagnosis, treatment of periodontal disease and make appropriate referrals to periodontists [10]. 

Over the years, several national periodontal societies across the world have been providing training to dentists to use this classification [11]. A number of studies have been conducted to assess the understanding, implementation and confidence of dental professionals in the use of 2017 classification [9, 10, 12–16]. The British Society of Periodontology conducted webinars to explain this new classification to UK dentists and dental hygienists so that they can implement it in their daily practice [17]. Whilst several studies have been conducted in Europe and America, there is a paucity of studies exploring the use of periodontal disease classification in Asian countries. Therefore, the present study was carried out as a pilot study to evaluate the diagnostic skills and self-perceived confidence of dentists and dental students in using the 2017 periodontal disease classification in Asian countries.

## Methods 

### Research ethics

This research as conducted in accordance with the Declaration of Helsinki ethical principles for medical research involving human subjects, including research on identifiable human material and data. Ethical approval of this study was approved by the institutional review board at university of Qatar (QU-IRB 1849-EA/23, dated 4th May 2023). Participation was voluntary and all data were recorded and processed anonymously. All participants provided their consent online before starting the survey.

### Study design

The present cross-sectional analytic study was conducted using an online questionnaire encompassing four periodontitis cases diagnosed based on clinical and radiographic information provided. The participants also asked to provide demographic information about their age, gender, location (city and country) and their current professional role.

### Setting

The study was conducted online using Google Forms (www.google.co.uk/forms/about).

### Participants

The target population included general dentists, periodontics specialist, other specialists, post graduate residents in periodontics and other specialties, and undergraduate students in Dentistry.

### Sample size calculation

The sample size for this study was determined G*Power software (version 3.1) [18]. In the planned analyses the sample size required to maintain a power of 0.8, with α = 0.05, and detect medium effects (f = 0.25–0.30) was calculated to be between *n* = 224 and *n* = 320 for the largest multifactorial designs. The achieved sample size of *n* = 314 therefore provides sufficient statistical power for these and the models, as well as for the correlational analyse conducted.

### Sampling technique

Purposive, non-probability sampling technique was used to recruit participants in the professional networks of the research team members. The study targeted participants based in five countries including India, Pakistan, Lebanon, United Arab Emirates (UAE) and Yemen.

### Data collection instrument

A questionnaire was created based on four periodontal cases which included patient’s relevant medical and dental history, intra-oral examination and periodontal examination including full mouth plaque score, bleeding on probing, furcation involvement according to Glickman’s Classification, mobility, periodontal charting (clinical attachment loss, gingival margin, probing pocket depth). A panoramic radiograph, a full set of periapical radiographs, and intra oral clinical images were also provided for each case.

### Data collection

A total of 500 participants were invited through email to respond to an online survey questionnaire using google forms. Participants were provided with an information sheet explaining the purpose and scope of the study. Prior to accessing the link, participants were required to provide an online consent confirming that their participation in the study was voluntary and they agreed to processing of their responses anonymously. Participants were asked to provide demographic information related to their age, gender, location and current status. Participants were asked to diagnose the cases according to 2017 classification of periodontal diseases. In addition, the participants were asked to indicate the level of confidence in their diagnosis on a scale from 1 to 10 ( 1 Not confident and 10-fully confident). All responses were recorded anonymously, and it was not possible to identify individual participants.

### Data analysis

Data analysis was conducted using the R statistical environment for Windows [19]. Pearson correlation coefficients were used where mean accuracy and confidence provided continuous variables, and analyses of variance were used to compare mean accuracy and confidence ratings between demographic groups.

## Results

A total of 314 participants provided their responses including 192 females and 120 males. Two participants withheld information regarding their gender and were included from subsequent analysis. The distribution of the participants by Age Group and gender is shown in Table 1.

**Table 1:** Participants by gender and age group

	Female		Male		Total^a^	
Age Group	n	%	n	%	n	%
18–25	86	44.79	38	31.67	124	39.74
26–35	83	43.23	42	35	125	40.07
36–45	16	8.33	27	22.5	43	13.78
Above 45	7	3.65	13	10.83	20	6.41
Total	192	100	120	100	312	100

^a^*n* = 2 Gender ‘Withheld or Omitted’ excluded from this table and subsequent analysis of Gender

The location and professional role of the participants are summarised in Tables 2 and 3 respectively.

**Table 2:** Participants by location

Location^a^	Number (*N*)	Percentage(%)
India	176	56.05
Lebanon	7	2.23
Pakistan	40	12.74
UAE	22	7.01
Yemen	41	13.06

^a^Groups of *n* ≤ 5 omitted

**Table 3:** Participants by professional role

Role^a^	*n*	%
General Dental Practitioners	82	26.11
Postgraduate Residents - Other	21	6.69
Postgraduate Residents in Periodontics	22	7.01
Specialists in Periodontics	64	20.38
Specialists Other	29	9.24
Undergraduate Dental Students	88	28.03

^a^Groups of *n* ≤ 5 omitted

The mean (M) diagnostic accuracy (%) by cases is shown in Table 4, and diagnostic interaction between demographic factors and diagnostic accuracy is summarized in Table 5.

**Table 4:** Mean diagnostic accuracy by case

Case	M	SD	*n*
Case1	43.31	49.63	314
Case2	15.61	36.35	314
Case3	22.61	41.90	314
Case4	23.57	42.51	314
Overall	26.27	44.03	1256

**Table 5:** Mean diagnostic accuracy by demographic factors

Age Group	M	SD	*n*
18–25	9.68	29.68	124
26–35	19.84	40.04	126
36–45	18.60	39.37	43
Above 45	19.05	40.24	21
Gender			
Female	15.63	36.40	192
Male	15.83	36.66	120
Location			
India	12.50	33.17	176
Other	19.57	39.81	138
Professional Role			
General Dental Practitioners	50.00	50.31	82
Specialists in Periodontics	57.81	49.78	64
Undergraduate Dental Students	25.00	43.55	88
Other	45.00	50.06	80

### Diagnostic accuracy by case and demographic factors

To compare the effects of demographic factors on diagnosis accuracy by case, a series of repeated measures ANOVAs were conducted, with accuracy, coded 0 (Incorrect) and 1 (Correct), as the outcome variable, Case (1,2,3,4) as a repeated measures factor, and, separately, Age Group, Gender, Location, and professional role included as additional factors. Coding accuracy of diagnosis in this way provides mean accuracies for each Case x Factor Level group that represent the proportion of correct diagnoses.

Age Group consisted of four levels; 18–25, 26–35, 36–45, and > 45. Given the small number of ‘Withheld or Omitted’ responses for Gender, these respondents were excluded from the ANOVAs. Location was collapsed into ‘India’ and ‘Elsewhere’ as India is the single largest location group. Professional role was reduced to ‘General Dental Practitioner’, ‘Specialist in Periodontics’, ‘Undergraduate Dental Student’, and Other (categories with < 40 respondents).

The results of the 4 Case x 4 Age Group ANOVA show a statistically significant difference in accuracy across Cases by Age Group (*F*_(9,924)_ = 2.304, *p* = 0.015), but no overall difference on accuracy by Age Group. ANOVA did not reveal any statistically significant main effect of Gender, or any interaction between Gender and Case on diagnosis accuracy. There was no statistically significant main effect of Location, or any interaction between Location and Case on diagnosis accuracy. However, the results showed a statistically significant difference in accuracy across Cases by professional role (*F*_(9,924)_ = 2.304, *p* = 0.005), and an overall difference on accuracy by professional role (*F*_(1,308)_ = 2.304, *p* = 0.012).

**Fig. 1:**
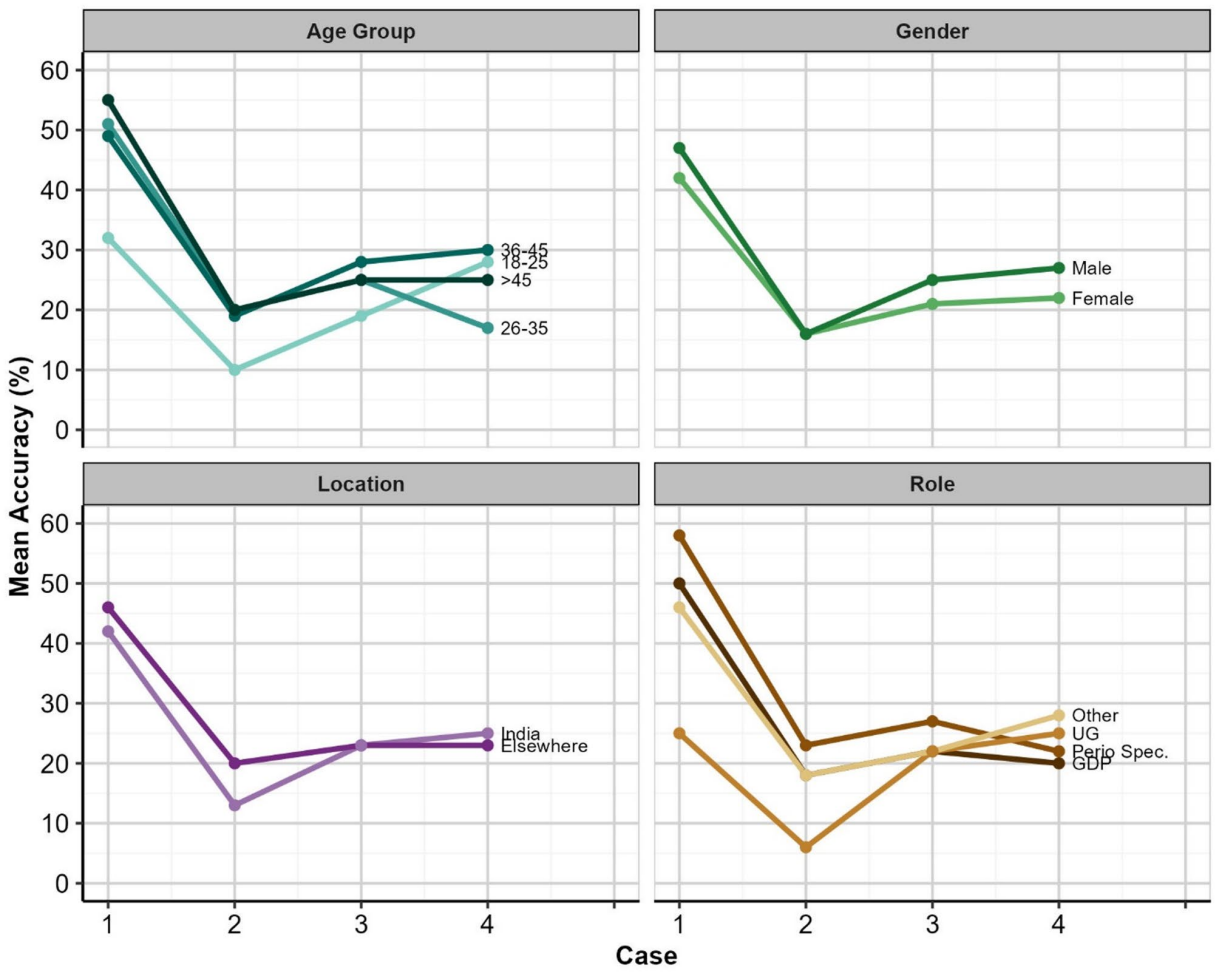
Mean Diagnosis accuracy (%) by Case and Demographic Group. *UG = Undergraduate students; Perio Spec = Periodontics specialists; GDP = General dental practitioners

Plots of the mean accuracy rates by case and demographic group are shown in Fig. 1. When considered together, the results suggested that the only statistically significant difference in accuracy is by Case (*F*_(3,849)_ = 12.714, *p* < 0.001), and that accuracy does not vary significantly as a function of Age Group, Gender, Location, or professional role.

### Confidence by case and demographic factors

**Fig. 2:**
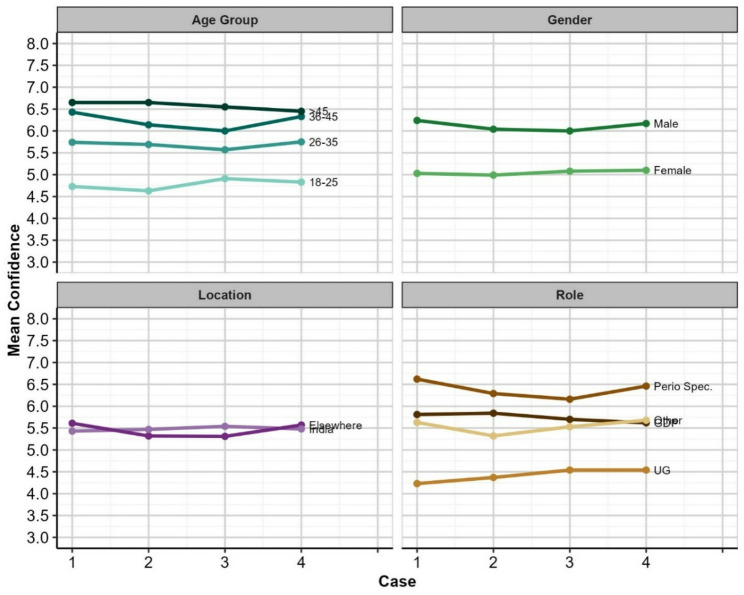
Mean Confidence rating (0–10) by Case and Demographic Group

The same analysis approach was used for Confidence ratings as for diagnosis accuracy, though with Confidence being treated as a continuous variable, such that means represent average confidence. ANOVA showed a statistically significant difference in confidence across Age Groups (*F*_(1,291)_ = 6.356, *p* < 0.001), but no interaction between Age Group and case. The results also showed a statistically significant difference in confidence across Genders (*F*_(1,293)_ = 13.747, *p* < 0.001), but no interaction between Gender and case. No statistically significant effect on confidence ratings by Location, or any interaction between location and case was observed. In regard to professional role, ANOVA showed a statistically significant difference in confidence across professional roles (*F*_(1,291)_ = 8.731, *p* < 0.001), but there was no interaction between professional role and case. Plots of the mean accuracy rates by case and demographic group are shown in Fig. 2. When considered together,, there appears to be no significant impact of these demographic factors on confidence ratings.

### Diagnostic accuracy by confidence

Whilst confidence varies little across cases within each demographic group, there is an overall positive association between mean accuracy and mean confidence ratings; *r*_(307)_ = 0.358, *p* < 0.001. Correlations between mean accuracy and mean confidence by demographic subgroups are shown in Table 6. These results suggest confidence is positively correlated with accuracy, to a medium degree, and that whilst it doesn’t always reach the threshold for statistical significance, this relationship holds across all subgroups.

**Table 6:** Correlation coefficients for the relationship between mean diagnosis accuracy and mean confidence by demographic subgroup

Factor	Level	*r* ^a^	*p*
Age Group	36–45	0.366	0.016
	26–35	0.374	< 0.001
	18–25	0.288	0.001
	Above 45	0.432	0.051
Gender	Male	0.423	< 0.001
	Female	0.304	< 0.001
Location	India	0.368	< 0.001
	Elsewhere	0.345	< 0.001
Professional role	Specialists in Periodontics	0.372	0.002
	General Dental Practitioners	0.281	0.011
	Undergraduate Dental Students	0.164	0.136
	Other	0.485	< 0.001

^a^Cohen’s effect size thresholds for *r*: small = 0.1, medium = 0.3, large = 0.5

## Discussion

The 2017 classification of periodontal diseases is widely used by dentists and dental hygienists based in Europe, America, and Australia and published literature indicates varying levels of confidence in the application of these guidelines in clinical practice. However, less information is available about the understanding and application of these guidelines by dentists in Asia and the current study aims to address this gap in the literature. Participants in the current study were from five different countries and included dentists in a wide range of professional roles including general dental practitioners and specialists as well as postgraduate and undergraduate dental students.

Previous studies in the United Kingdom indicate a high level of implementation of the 2017 guidelines in general dental practice settings with nearly 92% patients being diagnosed based on the latest classification system [20]. Similarly, there is evidence that the 2017 classification system has been widely adopted in undergraduate dental education in developed countries and its use has been reported by dental schools [10, 13, 21, 22]. Similarly, dental hygiene (DH) education programs in developed countries have also incorporated the guidelines in their clinical education. A study involving dental hygiene (DH) program directors reported 91% of DH education programs in United States, Canada, Australia had integrated the new staging and grading system into their curricula [23]. However, no comparable studies are reported from Asian countries. The regulatory frameworks in most Asian countries do not mandate the dentists to follow these guidelines and there is lack of clarity regarding adoption of periodontal disease classification system in Asian dental schools.

In the current study, the highest mean score for diagnostic accuracy and confidence was observed amongst specialists in periodontics with a mean score of 57.81 (± 49.78). The mean scores in other groups of dentists (general dentists, and other dental specialists) as well as dental students were lower. These results are not surprising as periodontal specialists are more likely to use these guidelines compared to other specialists and general dental practitioners [24, 25]. A previous study on general dental practitioners in Europe also identified gaps in their understanding and ability to apply periodontology guidelines for diagnosis of periodontitis [14]. The study showed that clinicians are better at correctly discriminating more advanced stages of periodontitis (better accuracy for stage III and IV compared to stage I and II) but have difficulties in discriminating between stage III and IV. In our study, the mean diagnostic was highest for case 1 (generalized periodontitis stage 3 grade B) followed by case 3 (localized stage 4 grade B) and case 4 (localized periodontitis stage 3 grade C). The lowest diagnostic accuracy was observed for case 2 (generalized periodontitis stage IV grade C). Since no cases with stage 1 or 2 were included in the current study, it was not possible to determine a similar trend.

Dental student participants in the current study showed the lowest diagnostic accuracy and a mean score of 25.00(± 43.55) was observed. The results are comparable to a similar study on dental students at two school in the United States which reported a diagnostic accuracy ranging from 25.2% to 27.6% amongst the participants [13]. However, another study reported higher diagnostic accuracy amongst dental students with a mean score of 50.0 amongst dental students in the second, third, and fourth years [22]. A study involving undergraduate dental students, orthodontic and periodontics trainees showed superior diagnostic skills of periodontal trainees compared to dental students and orthodontic trainees. These results are in accord with the findings of the current study [10]. In a recent study on dental hygienists in Sweden, it has been reported that nearly 70% Swedish hygienists use the 2017 classification system in their clinical practice but only one third were confident in application of these guidelines [16]. 

Challenges in the use of 2017 classification system have been highlighted by dentists and dental hygienists in previous studies. A study on dental practitioners showed that risk factor identification for periodontal disease was difficult for the participants including those with a background in periodontics [24]. A study on dental hygienists, found that the participants reported the classification system was difficult to apply in clinical practice and is considered time-consuming [16]. Clinical educators in periodontology need to identify appropriate strategies to facilitate an improved understanding of the periodontology guidelines amongst students and clinicians. Such strategies would broadly hinge on a sustained effort to provide case-based learning in clinical and non-clinical settings. Additionally, use of flow charts has been reported to improve the accuracy of diagnosing periodontal conditions in academic settings, especially amongst less experienced students [22]. Moreover, computer-based digital software can also assist dentists in achieving an accurate diagnosis of staging and extent of periodontitis [26]. 

Given the participants in this study were confined to five Asian countries, it may not be appropriate to generalize the findings to Asian dentists more broadly. The global burden of periodontal disease is significant and particularly high in Asian countries which constitute over half of the world’s population [27]. Four Asian countries, namely India, China, Indonesia and Pakistan are amongst the top five most populous countries in the world along with America [28]. The dental profession represents a global fraternity and dentists in less developed countries need to be supported to provide the most optimal preventive and clinical care to their communities to reduce the burden of periodontal disease and achieve improvements in clinical outcomes. There appears to be a need to have representation of Asian societies such as the Asian Pacific society of periodontology in developing and implementing updates to classification of periodontal disease. Notwithstanding the limitations of a representative sample from Asian countries, these results of the current study further exploration of implementation of periodontal disease classification in the participating countries using a larger and stratified sample.

Limitations:

There are several limitations of the current study that need to be acknowledged. Firstly, the study sample only involved 312 participants. Although the sample size met the requirements of power analysis, it is not representative of the wider population of dentists in Asian countries. Therefore, the findings are only applicable to the participants in this pilot study and should not be generalized to the wider population. Second, the study assessed the diagnostic accuracy and confidence of the participants using only four cases. Additional cases in the study along with assessment of treatment planning skills could have facilitated a better case mix and added more value to the findings. Moreover, the study did not investigate how many participants actually implemented the 2017 guidelines in their clinical practice. as the responses were collected through an online survey, it is possible that participants sought help from external sources such as online platforms, books, journals, or colleagues when completing the questionnaires, which represents a common limitation for online questionnaires. Given the low diagnostic accuracy of the participants in this study, this does not appear to be a high risk for the current study, though it cannot be entirely ruled out.

It is recommended that future studies involving a larger sample size stratified to dentists with different professional backgrounds and experience and dental students at different stages of education are conducted. Use of a mixed methods approach may provide further insights into the use of 2017 guidelines in clinical practice amongst Asian dentists and dental students and also explore the barriers they may face in implementing these guidelines in routine clinical care of their patients.

## Conclusion

The study shows the diagnostic accuracy and confidence was highest amongst periodontics specialists followed by general dentists and undergraduate students. Within the confines of the sample, participants demonstrated suboptimal diagnostic accuracy. Given the sample size, the findings are only applicable to the participants in this study and cannot be generalized to the broader population of dentists in Asian countries.

## Supplementary Information

Supplementary Material 1.

## Data Availability

The underlying raw data is available from the corresponding author at a reasonable request.

## References

[1] Sanz M, Marco del Castillo A, Jepsen S, et al. Periodontitis and cardiovascular diseases: consensus report. J Clin Periodontol. 2020. 10.1111/jcpe.13189. 32011025 10.1111/jcpe.13189 PMC7027895

[2] Wiebe CB, Putnins EE. The periodontal disease classification system of the American academy of Periodontology–an update. J Canad Dent Assoc. 2000;66(11):594–7. 11253351

[3] Chapple ILC, Mealey BL, Van Dyke TE, et al. Periodontal health and gingival diseases and conditions on an intact and a reduced periodontium: consensus report of workgroup 1 of the 2017 world workshop on the classification of periodontal and Peri-Implant diseases and conditions. J Periodontol. 2018. 10.1002/JPER.17-0719. 29926944 10.1002/JPER.17-0719

[4] Highfield J. Diagnosis and classification of periodontal disease. Aust Dent J. 2009. 10.1111/j.1834-7819.2009.01140.x. 19737262 10.1111/j.1834-7819.2009.01140.x

[5] Armitage GC. Development of a classification system for periodontal diseases and conditions. Ann Periodontol. 1999. 10.1902/annals.1999.4.1.1. 10863370 10.1902/annals.1999.4.1.1

[6] Caton G, Armitage J, Berglundh G, Chapple T, Jepsen ILC, Kornman SS, Mealey KL, Papapanou B, Sanz PN, Tonetti MS M. A new classification scheme for periodontal and peri-implant diseases and conditions – Introduction and key changes from the 1999 classification. J Clin Periodontol. 2018. 10.1111/jcpe.12935. 29926489 10.1111/jcpe.12935

[7] Berglundh T, Armitage G, Araujo MG, et al. Peri-implant diseases and conditions: consensus report of workgroup 4 of the 2017 world workshop on the classification of periodontal and Peri-Implant diseases and conditions. J Periodontol. 2018. 10.1002/JPER.17-0739. 29926955 10.1002/JPER.17-0739

[8] Papapanou PN, Sanz M, Buduneli N, et al. Periodontitis: consensus report of workgroup 2 of the 2017 world workshop on the classification of periodontal and Peri-Implant diseases and conditions. J Periodontol. 2018. 10.1002/JPER.17-0721. 29926951 10.1002/JPER.17-0721

[9] Dietrich T, Ower P, Tank M, et al. Periodontal diagnosis in the context of the 2017 classification system of periodontal diseases and conditions – Implementation in clinical practice. Br Dent J. 2019. 10.1038/sj.bdj.2019.3. 30631188 10.1038/sj.bdj.2019.3

[10] Abou-Arraj RV, Kaur M, Alkhoury S, Swain TA, Geurs NC, Souccar NM. The new periodontal disease classification: level of agreement on diagnoses and treatment planning at various dental education levels. J Dent Educ. 2021. 10.1002/jdd.12636. 33955000 10.1002/jdd.12636

[11] Hellyer P. Teaching the new periodontal disease classification. Br Dent J. 2021. 10.1038/s41415-021-3147-0. 34117431 10.1038/s41415-021-3147-0

[12] Oh SL, Yang JS, Kim YJ. Discrepancies in periodontitis classification among dental practitioners with different educational backgrounds. BMC Oral Health. 2021. 10.1186/s12903-020-01371-5. 33482794 10.1186/s12903-020-01371-5 PMC7821642

[13] Gandhi KK, Katwal D, Chang J, Blanchard S, Shin D, Maupome G, Eckert GJ, John V. Diagnosis and treatment planning using the 2017 classification of periodontal diseases among three dental schools. J Dent Educ. 2022. 10.1002/jdd.12964. 35644870 10.1002/jdd.12964

[14] Marini L, Tonetti MS, Nibali L, et al. The staging and grading system in defining periodontitis cases: consistency and accuracy amongst periodontal experts, general dentists and undergraduate students. J Clin Periodontol. 2021. 10.1111/jcpe.13406. 33260273 10.1111/jcpe.13406

[15] Tonetti MS, Sanz M. Implementation of the new classification of periodontal diseases: Decision-making algorithms for clinical practice and education. J Clin Periodontol. 2019. 10.1111/jcpe.13104. 30883878 10.1111/jcpe.13104

[16] Malmqvist S, Strandberg P, Victorin I, Boberg E, Johannsen A. The new system for classification of periodontal and peri-implant disease: A questionnaire study of implementation by Swedish dental hygienists. Int J Dent Hyg. 2024;23(3):625–31. 10.1111/idh.12816. 38721706 10.1111/idh.12816 PMC12371314

[17] Dorri M. Periodontal diseases: new classification for periodontal diseases. Br Dent J. 2018;225:686. 10.1038/sj.bdj.2018.941. 30361597 10.1038/sj.bdj.2018.941

[18] Faul F, Erdfelder E, Buchner A, Lang A-G. Statistical power analyses using G* power 3.1: tests for correlation and regression analyses. Behav Res Methods. 2009;41:1149–60. 10.3758/BRM.41.4.1149. 19897823 10.3758/BRM.41.4.1149

[19] R: The R Project for Statistical Computing. https://www.r-project.org/. Accessed 24 Nov 2023.

[20] Claydon N, Thomas DW, Adams RJ, West N, Hodge S. BSP implementation of the 2017 classification of periodontal diseases: a practice retrospective. Br Dent J. 2022. 10.1038/s41415-022-5220-8. 36434084 10.1038/s41415-022-5220-8

[21] Kakar A, Blanchard S, Shin D, Maupomé G, Eckert GJ, John V. Periodontal diagnosis and treatment planning – An assessment of the Understanding of the new classification system. J Dent Educ. 2022. 10.1002/jdd.13037. 35830257 10.1002/jdd.13037

[22] Parsegian K, Ayilavarapu S, Patel T, Henson HA, Angelov N. Flowcharts improve periodontal diagnosis by dental and dental hygiene students. Canad J Dent Hyg. 2021;55(3):137–47. 34925514 PMC8641549

[23] Aboalsaud KM, Foster NL, Yu SH, Sweier DG, Rulli D. Periodontal staging and grading: an international dental hygiene education survey. Int J Dent Hyg. 2023. 10.1111/idh.12627. 36098686 10.1111/idh.12627

[24] Oh S-L, Yang JS, Kim YJ, Oh SL, Yang JS, Kim YJ. (2021). Discrepancies in periodontitis classification among dental practitioners with different educational backgrounds. BMC Oral Health. 21(1):39. 10.1186/s12903-020-01371-5 33482794 10.1186/s12903-020-01371-5 PMC7821642

[25] Ravidà A, Travan S, Saleh MHA, Greenwell H, Papapanou PN, Sanz M, Tonetti M, Wang HL, Kornman K. Agreement among international periodontal experts using the 2017 world workshop classification of periodontitis. J Periodontol. 2021. 10.1002/JPER.20-0825. 34545953 10.1002/JPER.20-0825

[26] Meir H, Miller H, Saminsky M, Huang Y, Levin L, Slutzkey G. Computer-assisted periodontal classification vs manual classification among dental students: a comparative pilot study. Quintessence Int (Berl). 2023. 10.3290/j.qi.b4225983. 10.3290/j.qi.b4225983 37477040

[27] Corbet EF. Periodontal diseases in Asians. J Int Acad Periodontol. 2006;8(4):136–44. 17042169

[28] World Population by Country 2024. (Live). Available at: https://worldpopulationreview.com/. Accessed 6 Sept 2024.

