# Supplementary material for: Periodontal precision: diagnostic skills and confidence of dentists in Asian countries in applying the 2017 EFP/AAP periodontal disease classification- a cross-sectional pilot study
Source: BMC Oral Health. 2025 Dec 5;25:1882. doi: 10.1186/s12903-025-07030-x (PMC12683845; doi:10.1186/s12903-025-07030-x)
Supplement: Supplementary file 1 — Supplementary Material 1. [file 12903_2025_7030_MOESM1_ESM.pdf]

Classification of Periodontal Diseases (2017): Application and Confidence among Dental Professionals

The aim of this study is to evaluate the application and confidence among dental professionals to diagnose the periodontal diseases as per 2017 World workshop classification. This study is done by a group of dental academics based at Qatar University, Plymouth University and Canada . You are provided with 4 cases. Kindly provide your diagnosis of each case as per the classification of periodontal disease (2017) All results will be analyzed anonymously, and individual participants will not be identified.

* Indicates required question

1. Do you provide your consent to participate in this study ? *

☐ Yes
☐ No

Demographics

2. **Age (Years)**

☐ 18-25

☐ 26-35

☐ 36-45

☐ Above 45

3. **Gender**

☐ Female

☐ Male

☐ Other

☐ Prefer not to say

4. **Location** (City and Country) *

____

5. **Current status**

☐ General Dental Practitioner

☐ Specialist in Periodontics

☐ Specialist Other

☐ Postgraduate Resident in Periodontics

☐ Postgraduate Resident - Other

☐ Undergraduate Dental Student

☐ Other: _______________________

Patient Cases

Please read the case histories of four cases and review attached Periodontal charting , images. You are requested to provide your diagnosis for each case and also indicate how confident you are with your diagnosis?

CASE 1

A 74 year old female presents with the following history:

Medical History :

- Hypothyroidism (Levothyroxine sodium)
- Hypertension (Amlodipine)
- Type II Diabetes Mellitus (on Insulin)

No Known Drug Allergy

Intraoral Examination:

- Moderately restored dentition.
- Porcelain fracture #25-27 bridge.

Periodontal Examination:

- Full Mouth Plaque Score: 72%
- Bleeding on Probing : 28%
- Furcation involvement (Glickman):
- Grade I # 17M/D, 47B
- Grade II # 16B/D, 17B, 27B/D, 36B/L, 37B/L, 46B/L, 47B
- Mobility (Miller):
- Grade I # 31, 32, 41, 42, 43

Periodontal Charting (CAL : Clinical Attachment Loss, GM: Gingival Margin , PPD : Probing Pocket Depth)

CAL	5 5 5	5 4 8	5 4 6	5 4 6	66 7	6 4 4	6 5 5	4 3 4	4 2 5	4 3 3	6 4 4	3 3 2		4 3 4
GM	1 2 1	2 23	1 21	1 2 1	2 3 3	2 2 1	2 3 2	1 2 1	0 1 0	0 1 0	1 2 1	0 10		1 1 1
PPD	5 3 4	3 25	425	4 2 5	4 3 4	4 2 3	4 2 3	3 1 3	4 1 5	4 2 3	5 2 3	32 2		3 2 3
	17	16	15	14	13	12	11	21	22	23	24	25	26	27
PPD	4 2 4	4 2 5	4 2 7	6 3 4	6 3 4	3 2 3	3 2 3	3 2 3	4 3 4	5 3 5	5 2 4	3 1 4		31 2
GM	0 2 0	0 2 0	0 1 0	0 1 0	0 0 0	0 0 0	0 0 0	0 0 0	0 0 0	0 0 0	0 0 0	1 1 1		1 1 1
CAL	4 4 4	445	437	64 4	6 3 4	3 2 3	3 2 3	323	4 3 4	5 3 5	5 2 4	4 2 5		4 2 3

CAL	5 3 6	5 5 6	524	634	443	333	444	645	434	335	435	443	655	475
GM	11 1	12 1	000	000	010	010	121	222	111	000	000	010	121	121
PPD	42 5	435	524	634	433	323	323	423	323	335	435	433	534	354
	47	46	45	44	43	42	41	31	32	33	34	35	36	37
PPD	33 4	434	534	425	523	323	321	223	313	324	424	424	5 26	4 2 4
GM	22 2	222	111	121	232	121	111	232	131	121	020	000	111	010
CAL	55 6	656	635	546	755	444	432	455	444	445	444	424	637	4 3 4

Radiographic and Clinical Images:

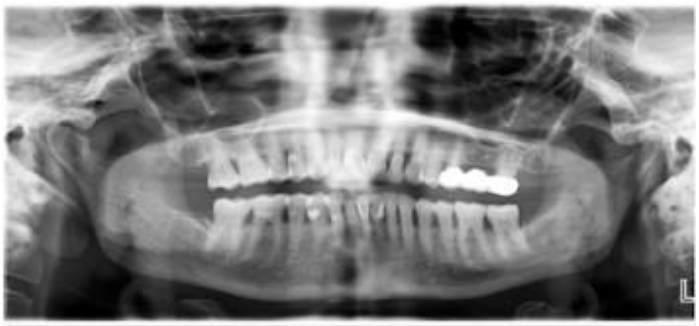

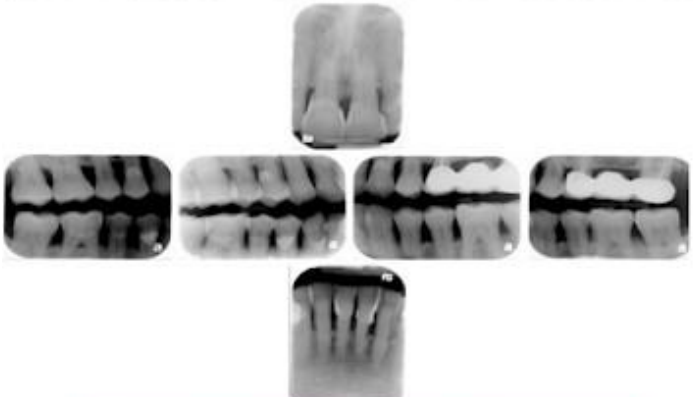

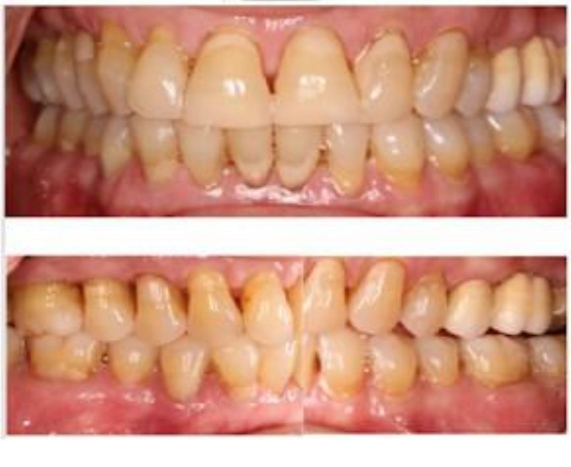

1A: *Based on the history , periodontal charting, clinical and radiographic presentation, what is the diagnosis of this case according to 2017 Classification of Periodontal Disease?*

Mark only one oval.

- Stage I Periodontitis Grade B
- Stage II Periodontitis Grade C
- Stage III Periodontitis Grade B
- Stage IV Periodontitis Grade A

1B: *How confident are you with your diagnosis?*

Mark only one oval.

	1	2	3	4	5	6	7	8	9	10	
Not Confident	○	○	○	○	○	○	○	○	○	○	Fully Confident

CASE 2

A 54 year old female patient presents with following history:

Medical History :

- No current medical conditions or medications reported by the patient.
- Penicillin allergy.

Intraoral Examination:

- Extensively restored dentition, multiple tipped/rotated teeth, poor crown margins.
- Bilateral mandibular tori.
- Mild attrition anterior dentition.
- NW excursive interferences #27, 36, 37, 17, 47.
- Fremitus #22.

Periodontal Examination:

- FMPS: 63%
- BOP: 60%
- Furcation involvement (Glickman):
- Grade I # 16D, 17M, 26B, 27B, 37B, 46B, 47B
- Grade II #27D, 36B/L, 37L, 47L
- Mobility (Miller):
- Grade I # 11, 14, 24, 26, 31, 32, 34, 35, 36, 41, 42, 44, 45
- Grade 2 # 12, 21, 22
- Mucogingival involvement (MGI):
- #35, 41, 42

Periodontal Charting (CAL : Clinical Attachment Loss, GM: Gingival Margin , PPD : Probing Pocket Depth)

CAL	4 4 5	9 7 5	3 4 7	3 5 6	2 5 7	6 5 3	3 2 3	6 4 5	7 5 6	5 5 4	6 5 7	3 3 4	3 5 7	6 4 4
GM	0 0 0	3 3 2	0 1 1	0 2 0	0 2 2	3 1 0	0 0 0	1 1 1	3 2 3	2 3 0	2 3 3	0 0 0	0 1 2	1 1 1
PPD	4 4 5	6 4 3	3 3 6	3 3 6	2 3 5	3 4 3	3 2 3	5 3 4	4 3 3	3 2 4	4 2 4	3 3 4	3 4 5	5 3 3
	17	16	15	14	13	12	11	21	22	23	24	25	26	27
PPD	4 3 6	5 4 4	3 7 7	4 4 7	3 4 5	4 3 4	4 3 3	6 3 4	5 3 4	5 3 3	3 3 5	3 3 3	4 3 3	4 4 4
GM	0 0 2	1 1 1	0 0 0	0 0 0	0 1 2	2 1 1	1 0 0	1 1 1	3 3 3	2 1 0	0 1 0	0 0 0	0 0 0	0 0 0
CAL	4 3 8	6 5 5	3 7 7	4 4 7	3 5 7	6 4 5	5 3 3	7 4 5	8 6 7	7 4 3	3 3 5	3 3 3	4 3 3	4 4 4

CAL	5 5 4	5 5 6	3 3 3	3 3 3	7 4 4	3 5 3	3 4 3	3 4 2	4 6 2	4 3 4	4 3 3	3 3 5	6 5 8	5 4 3
GM	0 0 0	2 2 2	0 0 0	0 0 0	3 1 0	0 2 0	0 2 0	0 2 0	0 2 0	0 0 1	0 0 0	0 0 0	2 1 1	0 0 0
PPD	5 5 4	3 3 4	3 3 3	3 3 3	4 3 4	3 3 3	3 2 3	3 2 2	4 4 2	4 3 3	4 3 3	3 3 5	4 4 7	5 4 3
	47	46	45	44	43	42	41	31	32	33	34	35	36	37
PPD	4 3 4	4 4 4	3 3 2	3 2 5	4 2 3	3 2 3	3 2 3	2 2 3	3 3 4	4 2 3	4 3 4	3 3 4	4 4 9	5 4 8
GM	0 0 0	0 1 0	0 0 0	0 1 0	0 1 0	0 0 0	0 0 0	0 1 0	0 2 0	0 2 0	0 2 0	0 0 0	0 0 0	0 0 0
CAL	4 3 4	4 5 4	3 3 2	3 3 5	4 3 3	3 2 3	3 2 3	2 2 3	3 5 4	4 4 3	4 5 4	3 3 4	4 4 9	5 4 8

Radiographic and Clinical Images:

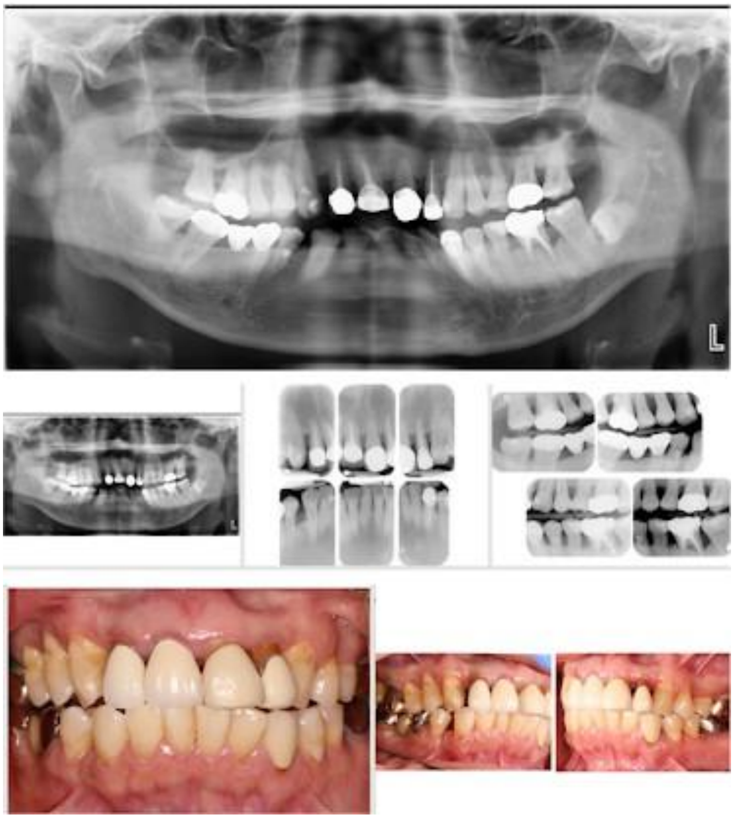

2 A: ***Based on the history , periodontal charting, clinical and radiographic presentation, what is the diagnosis of this case according to 2017 Classification of Periodontal Disease?***

Mark only one oval.

- Stage I Periodontitis Grade C
- Stage II Periodontitis Grade C
- Stage III Periodontitis Grade C
- Stage IV Periodontitis Grade C

2 B: ***How confident are you with your diagnosis?***

Mark only one oval.

	1	2	3	4	5	6	7	8	9	10	
Not Confident											Fully Confident

.

CASE 3

A 70 year old female presents with following history :

Medical History :

- Hypertension (Atenolol, Irbesartan, Nifedipine)
- Obstructive sleep apnea

Intraoral Examination:

- Generalized fibrotic gingiva
- Torus palatinus
- Fremitus #1.4, (working side interference)

Periodontal Examination:

- FMPS: 11%
- BOP: 16%
- Furcation involvement:
- Grade I # 26B, 37B
- Grade II # 26D
- Mobility:
- Grade I # 11, 14, 15
- Grade I+ # 12

Periodontal Charting (CAL : Clinical Attachment Loss, GM: Gingival Margin , PPD : Probing Pocket Depth)

CAL			5 5 5	6 5 8	6 4 4	4 5 11	6 4 6	4 2 3	3 2 3	3 2 3	3 3 4	4 4 5	5 3 9	
GM			2 3 1	1 2 0	0 1 0	0 0 0	0 0 0	0 0 0	0 0 0	0 0 0	0 1 0	0 1 0	0 1 1	
PPD			3 2 4	5 3 8	6 3 4	4 5 11	6 4 6	4 2 3	3 2 3	3 2 3	3 2 4	4 2 5	5 2 8	
	17	16	15	14	13	12	11	21	22	23	24	25	26	27
PPD			5 6 5	6 4 8	5 4 3	3 5 10	5 3 7	3 2 3	3 2 3	3 3 3	3 2 3	3 2 3	5 3 5	
GM			0 0 0	0 1 0	0 0 0	0 1 0	0 0 0	0 0 0	0 0 0	0 0 0	0 1 0	0 0 0	0 2 2	
CAL			5 6 5	6 5 8	5 4 3	3 6 10	5 3 7	3 2 3	3 2 3	3 3 2	3 3 3	3 2 3	5 5 7	

CAL			3 2 3	3 2 3	3 2 3	3 2 2	2 2 2	2 2 2	2 3 2	2 3 2	2 2 2	3 4 6		4 4 4
GM			1 0 0	0 0 0	0 0 0	0 0 0	0 0 0	0 0 0	0 1 0	0 1 0	0 0 0	0 2 3		1 2 1
PPD			3 2 3	3 2 3	3 2 3	3 2 2	2 2 2	2 2 2	2 2 2	2 2 2	2 2 2	3 2 3		3 2 3
	47	46	45	44	43	42	41	31	32	33	34	35	36	37
PPD			3 2 3	3 2 4	5 2 2	2 2 2	2 2 2	3 2 3	3 2 3	3 2 3	3 2 3	3 2 3		5 2 2
GM			2 1 0	0 0 0	0 0 0	0 0 0	0 0 0	0 0 0	0 0 0	0 0 0	0 0 0	0 0 1		1 1 1
CAL			5 3 3	3 2 4	5 2 2	2 2 2	2 2 2	3 2 3	3 2 3	3 2 3	3 2 3	3 2 4		6 3 3

Radiographic and Clinical Images :

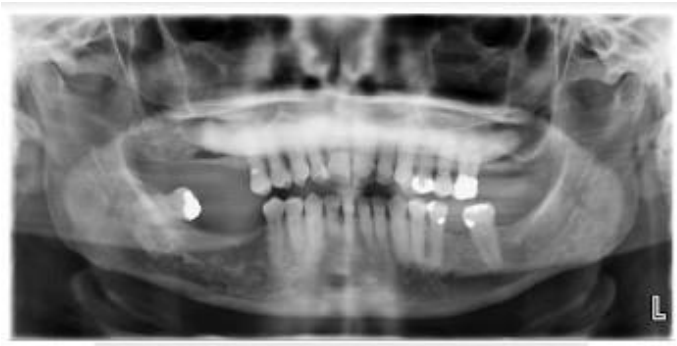

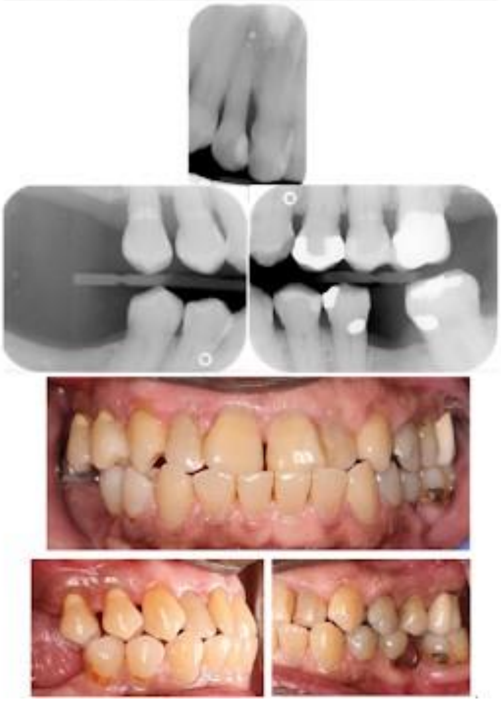

3 A: Based on the history , periodontal charting, clinical and radiographic presentation, what is the diagnosis of this case according to 2017 Classification of Periodontal Disease?

Mark only one oval.

- Stage I Periodontitis Grade C
- Stage IV Periodontitis Grade B
- Stage III Periodontitis Grade C
- Stage III Periodontitis Grade B

3 B: How confident are you with your diagnosis?

Mark only one oval.

	1	2	3	4	5	6	7	8	9	10	
Not Confident	○	○	○	○	○	○	○	○	○	○	Fully Confident

Case 4

A 58 year old male presents with following history:

Medical History :

- No current medical conditions or medications reported by the patient.
- Smoker ~4 packs/week.
- Previous reaction to penicillin (unsure of type).

Intraoral Examination:

- Several restorations.
- Mild crowding in mx/md arches
- #27 in infraocclusion, no response to endo-ice testing. Suppuration on probing of #27.

Periodontal Examination:

- FMPS: 81%
- BOP: 39%
- Furcation involvement (Glickman):
- Grade I # 17M/D, 18M, 38B/L, 46B
- Grade II #48L
- Grade III #27
- Mobility (Miller):
- Grade I # 27, 32

Periodontal Charting (CAL : Clinical Attachment Loss, GM: Gingival Margin , PPD : Probing Pocket Depth)

CAL	6 3 4		4 4 2	2 2 3	2 3 2	2 2 2	2 1 2	3 1 3	3 2 3	3 3 3	3 3 3	3 2 3	3 4 4	58 14
GM	1 1 1		2 2 0	0 1 0	0 2 0	0 0 0	0 0 0	0 0 0	0 0 0	0 1 0	0 1 0	0 0 0	0 1 0	0 2 2
PPD	5 2 3		2 2 2	2 1 3	2 1 2	2 2 2	2 1 2	3 1 3	3 2 3	3 2 3	3 2 3	3 2 3	3 3 4	56 12
	17	16	15	14	13	12	11	21	22	23	24	25	26	27
PPD	6 2 3		2 2 3	3 2 3	3 2 3	2 2 2	2 2 2	3 1 3	2 1 3	3 2 3	3 2 3	3 2 3	3 2 4	32 12
GM	2 2 2		2 1 0	0 0 0	0 0 0	0 0 0	0 0 0	0 0 0	0 0 0	0 0 0	0 0 0	0 0 0	2 1 0	0 1 0
CAL	8 4 5		4 3 3	3 2 3	3 2 3	2 2 2	2 2 2	3 1 3	2 1 3	3 2 3	3 2 3	3 2 3	5 3 4	33 12

CAL	4 2 5	4 2 5	4 2 3	3 2 3	3 3 3	2 5 4	3 3 3		5 4 5	5 4 4	3 2 3	3 2 3	3 2 3	3 2 3
GM	0 0 0	0 0 0	0 0 0	0 0 0	0 0 0	1 3 2	1 1 1		2 2 2	2 2 1	0 0 0	0 0 0	0 0 0	0 0 0
PPD	4 2 5	4 2 5	4 2 3	3 2 3	3 3 3	1 2 2	2 2 2		3 2 3	3 2 3	3 2 3	3 2 3	3 2 3	3 2 3
	47	46	45	44	43	42	41	31	32	33	34	35	36	37
PPD	4 2 3	3 2 3	3 2 3	3 2 3	3 2 3	3 2 3	2 1 2		2 1 2	2 2 3	3 2 3	3 2 3	3 2 3	3 2 3
GM	0 0 0	0 1 0	0 0 0	0 0 0	0 0 0	0 0 0	0 0 0		0 0 0	0 0 0	0 0 0	0 0 0	0 0 0	0 0 0
CAL	4 2 3	3 3 3	3 2 3	3 2 3	3 2 3	3 2 3	2 1 2		2 1 2	2 2 3	3 2 3	3 2 3	3 2 3	3 2 3

. ***Radiographic and Clinical Images:***

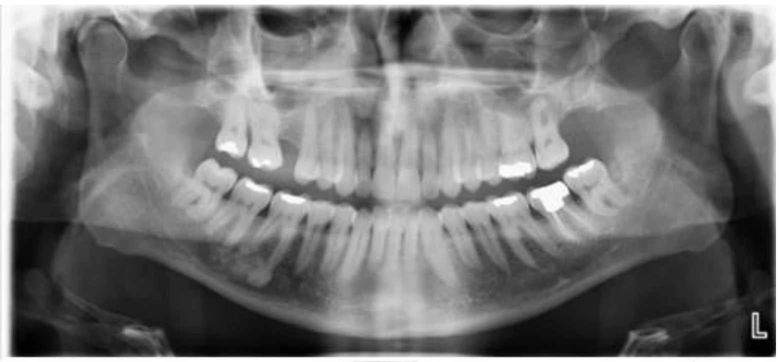

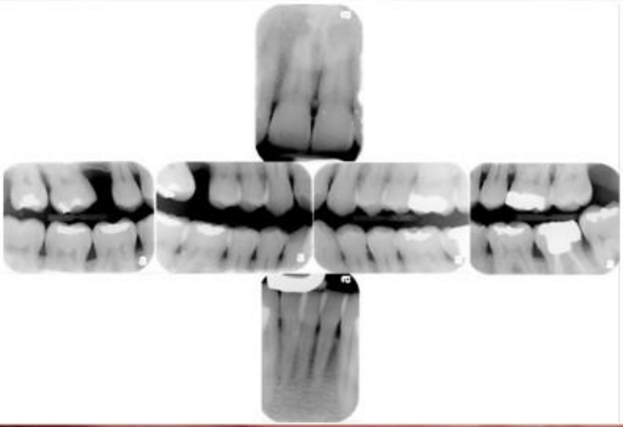

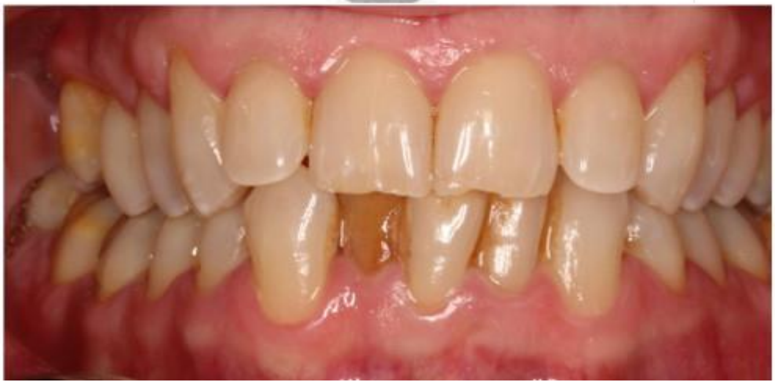

4 A: Based on the history , periodontal charting, clinical and radiographic presentation, what is the diagnosis of this case according to 2017 Classification of Periodontal Disease?

Mark only one oval.

- Stage III Periodontitis Grade B
- Stage III Periodontitis Grade C
- Stage II Periodontitis Grade A
- Stage II Periodontitis Grade C

4 B: How confident are you with your diagnosis?

Mark only one oval.

	1	2	3	4	5	6	7	8	9	10	
Not Confident											Fully Confident

This content is neither created nor endorsed by Google.

Google Forms
